# On the G-Protein-Coupled Receptor Heteromers and Their Allosteric Receptor-Receptor Interactions in the Central Nervous System: Focus on Their Role in Pain Modulation

**DOI:** 10.1155/2013/563716

**Published:** 2013-07-17

**Authors:** Dasiel O. Borroto-Escuela, Wilber Romero-Fernandez, Alicia Rivera, Kathleen Van Craenenbroeck, Alexander O. Tarakanov, Luigi F. Agnati, Kjell Fuxe

**Affiliations:** ^1^Department of Neuroscience, Karolinska Institutet, Retzius väg 8, 17177 Stockholm, Sweden; ^2^Faculty of Science, University of Malaga, 29080 Malaga, Spain; ^3^Laboratory of Eukaryotic Gene Expression and Signal Transduction (LEGEST), Ghent University-Gent, 9000 Ghent, Belgium; ^4^Russian Academy of Sciences, St. Petersburg Institute for Informatics and Automation, 193167 Saint Petersburg, Russia; ^5^IRCCS, Lido Venice, 41100 Venice, Italy

## Abstract

The modulatory role of allosteric receptor-receptor interactions in the pain pathways of the Central Nervous System and the peripheral nociceptors has become of increasing interest. As integrators of nociceptive and antinociceptive wiring and volume transmission signals, with a major role for the opioid receptor heteromers, they likely have an important role in the pain circuits and may be involved in acupuncture. The delta opioid receptor (DOR) exerts an antagonistic allosteric influence on the mu opioid receptor (MOR) function in a MOR-DOR heteromer. This heteromer contributes to morphine-induced tolerance and dependence, since it becomes abundant and develops a reduced G-protein-coupling with reduced signaling mainly operating via **β**-arrestin2 upon chronic morphine treatment. A DOR antagonist causes a return of the Gi/o binding and coupling to the heteromer and the biological actions of morphine. The gender- and ovarian steroid-dependent recruitment of spinal cord MOR/kappa opioid receptor (KOR) heterodimers enhances antinociceptive functions and if impaired could contribute to chronic pain states in women. MOR1D heterodimerizes with gastrin-releasing peptide receptor (GRPR) in the spinal cord, mediating morphine induced itch. Other mechanism for the antinociceptive actions of acupuncture along meridians may be that it enhances the cross-desensitization of the TRPA1 (chemical nociceptor)-TRPV1 (capsaicin receptor) heteromeric channel complexes within the nociceptor terminals located along these meridians. Selective ionotropic cannabinoids may also produce cross-desensitization of the TRPA1-TRPV1 heteromeric nociceptor channels by being negative allosteric modulators of these channels leading to antinociception and antihyperalgesia.

## 1. Introduction

The monoamine-peptide interactions have been of great interest [1]. How did they, in fact, interact at the molecular level? One possibility was that the monoamine and peptide signals became integrated through direct neuropeptide-monoamine receptor-receptor interactions in the plasma membrane. We began to test this hypothesis in 1980-1981 in membrane preparations of various Central Nervous System (CNS) regions and found that neuropeptides could modulate the binding characteristics, especially the affinity, of the monoamine receptors in a receptor subtype specific way [2, 3]. Thus, intramembrane receptor-receptor interactions did exist besides indirect actions via phosphorylation and changes in membrane potential. The results were in line with earlier findings by Limbird et al. in 1975, showing negative cooperativity in *β*-adrenergic receptors, which may be explained by the existence of receptor homodimers leading to orthosteric site-site interactions [4]. It was also clear that adapter proteins can be involved in mediating the receptor-receptor interactions in brain membranes [5, 6].

In this paper we give a brief overview of the modulatory role of receptor heteromers in the pain pathways in the CNS and in peripheral nociceptors. They seem to be integrators of nociceptive and antinociceptive wiring (WT) and volume transmission (VT) signals with a major role for the opioid receptor heteromers. Their relevance in the mechanisms for the antinociceptive actions of acupuncture will be discussed.

## 2. Primary Afferents of the Dorsal Horn Mediating Nociception

**Figure 1:**
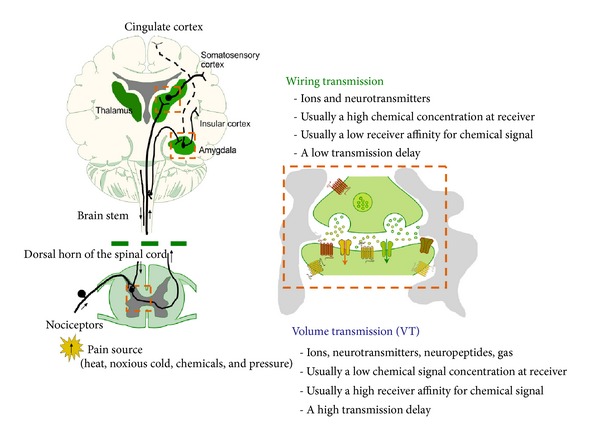
Pain pathways and their regulation through WT and VT signals integrated through receptor-receptor interactions in heteromers. (left) A schematic overview of the ascending main circuits mediating pain. When a noxious stimulus is encountered. Afferent nociceptors convey noxious information to projection neurons within the dorsal horn of the spinal cord. Neurotransmitters released here bind to and activate postsynaptic receptors on pain transmission neurons. In turn, the axons of pain transmission neurons ascend, predominantly contralaterally, to the brain and carry the information about the noxious stimulus to higher centers (somatosensory cortex via the thalamus with information about location and intensity of the painful stimulus or the insular cortices via connections in the brainstem (parabranchial nucleus) and amygdala within the affective component of the pain experience). The descending inhibitory pathways to the dorsal horn from the brainstem involving interalia the NA, 5HT, and DA pathways (see text) are also indicated. They exert antinociceptive actions in the pain circuits of the dorsal horn. (right) The diagram shows a few prominent of many possible mediators and cell-cell interactions in the spinal cord dorsal horn, thalamus, or amygdala. In these pain circuits opioid receptor containing heteromers may play a role in the modulation of pain transmission, offering novel targets for antinociceptive drugs. The enkephalin peptides (short distance diffusion) and b-endorphin (long distance diffusion) mainly operate via VT and likely modulate the pain circuits via receptor-receptor interactions in receptor heteromers built-up of synaptic protomers and of opioid receptor protomers. In this way synaptic transmission signals and VT signals become integrated giving a balance in nociceptive and antinociceptive signaling in the CNS. The descending inhibitory pathways to the dorsal horn involving inter alia the monoamine pathways also mainly communicate via VT (see text).

We have different types of nociceptors coming into the dorsal horn (primary afferent fibres). One major class includes medium diameter myelinated (A*δ*) afferents that mediate well-localized fast pain. The second class of nociceptor includes small diameter unmyelinated “C” fibers that mediate poorly localized, slow pain [7]. There exist a very precise laminar organization of the dorsal horn of the spinal cord. The unmyelinated, peptidergic C and myelinated A*δ* nociceptors terminate most superficially, synapsing upon large projection neurons in lamina I and interneurons located in outer lamina II. Thus, primary afferent nociceptors convey noxious information to projection neurons within the dorsal horn of the spinal cord (Figure 1, left).

## 3. Pain Pathways and Their Regulation through WT and VT Signals Integrated through Receptor-Receptor Interactions in Heteromers

A subset of these projection neurons transmits information to the somatosensory cortex via the thalamus, providing information about the location and intensity of the painful stimulus [7]. Other projection neurons reach the cingulate and insular cortices via relay stations in the lower brainstem (parabrachial nucleus) and amygdala, contributing to the emotional component of the pain experience (Figure 1, left). This ascending information also passes on to neurons of the rostral ventral medulla, including the raphe and pararaphe area and the periaqueductal gray of the midbrain to activate descending feedback systems like distinct bulbo-spinal serotonin (5-HT) and noradrenergic (NA) neurons [8] that regulate the pain transmission output from the spinal cord, especially the lateral paragigantocellular reticular 5-HT neurons may be involved in this process.

This important descending inhibitory control of nociception in the dorsal horn of the spinal cord (Figure 1, left) involves especially the periaqueductal gray-rostral ventromedial medulla, the dorsal reticular nucleus of the medulla, and the ventrolateral medulla [9]. The descending bulbo-spinal NA and 5-HT neuron systems [10–12] play a substantial role [13–15]. Noxious stimulation excites the 5-HT neurons of the lateral paragigantocellular reticular and raphe magnus nuclei [16], building up group B3 of Dahlstroem and Fuxe [10] and these 5-HT neuron systems produce strong analgesia and the former also cardiovascular activation. Also descending projections to the dorsal horn from DA neurons in the posterior hypothalamus participate in control of pain [17]. The major descending inhibitory bulbo-spinal systems are under the control of high densities of mu opioid receptors [18, 19]. Blockade of the opioid receptors in the medullary reticular nucleus dorsalis prevents analgesia produced by diffuse noxious inhibitory controls [20]. The anti-nociceptive actions of the descending inhibitory pathways in the spinal cord involves the indirect activation of opioid receptors in the dorsal horn, especially in the superficial layers. The major mode of communication in the descending inhibitory monoamine pathways of nociception is extrasynaptic volume transmission involving monoamine diffusion in the um range [21] and activation of their extrasynaptic receptors located on the nociceptors and their target nerve cells mainly found in the superficial layers in the dorsal horn. However, wiring transmission also exists in these descending systems through formation of monoamine synapses. When neuropeptides are released from these descending inhibitory pathways long distance volume transmission can develop in the order of 0.1–1 mm and also involve CSF volume transmission provided the peptides do not undergo rapid degradation [22].

In these pain circuits we will describe how opioid receptor containing heteromers may play a role in the modulation of pain transmission, offering novel targets for antinociceptive drugs. They may be involved in conveying the antinociceptive actions of acupuncture [23] since inter alia electroacupuncture has been shown to increase transcription and translation of enkephalins in the rostral ventrolateral medulla of rats, a region involved in regulation of not only circulation and respiration but also pain [24]. The enkephalin peptides (short distance diffusion) and *β*-endorphin (long distance diffusion) mainly operate via VT [25–27] and likely modulate the pain circuits via receptor-receptor interactions in receptor heteromers built-up of synaptic protomers and of opioid receptor protomers. In this way synaptic transmission signals and VT signals become integrated giving a balance in nociceptive and antinociceptive signaling in the CNS (Figure 1).

## 4. Principal Features of the Receptor Heteromers

As mentioned in the introduction, the concept of intramembrane receptor-receptor interactions in receptor heteromers was born in the analysis of neuropeptide-monoamine receptor-receptor interactions in 1980-81. The concept of G protein-coupled receptor (GPCR) heteromers was later confirmed in 1998-1999 by studies reporting that two non-functional GPCR monomers, gamma amino butyric acid (GABA)B1 and GABAB2 receptors, can assemble in a signalling heterodimer, the GABAB receptor at the cell surface [28, 29]. 

## 5. The Receptor-Receptor Interaction Toolbox

### 5.1. Fluorescence and Bioluminescence Resonance Energy Transfer Methods

Different resonance energy transfer methods have been used to study the existence of GPCR heteromers [30–33]. Fluorescence resonance energy transfer (FRET) method using for example, the CFP-YFP pair in the labeling of two receptors. If the receptors physically interact the distance is short enough (10 nm or less) to allow energy transfer from the donor CFP on one receptor to the acceptor YFP on the other receptor, thus a FRET signal develops from YFP. At the cell surface in living cells, the time-resolved FRET (TR-FRET) method have been developed to study receptor heteromers, by conjugating donor and acceptor fluorophore molecules to antibodies against each protomer in the heteromer of interest. TR-FRET is based on the engagement of a resonance energy transfer process between a lanthanide, such as europium (Eu^3+^) or terbium (Tb^3+^) cryptate, as a donor molecule and a compatible acceptor chromophore, such as alexafluor 647 or allophycocyanin [30].

Principle of the detection of GPCR heterodimerization using the bioluminescence resonance energy transfer BRET method is similar to the FRET method except a *Renilla luciferase*-GFP_2_/YFP pair is used in the tagging of the two receptors, in the presence of the substrate h-coelenterazine or coelenterazine-400 on which *Renilla luciferase* acts to produce through oxidation a bioluminescent signal. Then the energy transfer between the generated luminescence and YFP or GFP_2_ occurs when the distance between these proteins is less than 10 nm leading to a fluorescence emission from YFP or GFP_2_ [21].

On the other hand, the principle of bimolecular fluorescence complementation (BiFC) methods is based on the complementation of the N-terminal and C-terminal fragment of a fluorescent protein (e.g., YFP) [34]. After interaction of the tagged receptors, the protein fragments reconstitute a functional fluorescent protein interpreted as a result of GPCR heteromer formation.

A drawback of these methods is the fact that they involve the ectopic expression and/or overexpression of the fusion receptors, thereby, sometimes promoting the formation of artefacts. It is therefore advisable, whenever possible, to consider the physiological expression levels of the receptor pairs under study.

### 5.2. In Situ Proximity Ligation Assay

However, despite extensive experimental results supporting the formation of GPCR heteromers in heterologous systems (mainly by BRET and FRET methodologies), the existence of such heteromers in the CNS and other tissues remains largely unknown, mostly because of the lack of appropriate methodology. Recently, a well-characterized in situ proximity ligation assay (in situ PLA) has been adapted to confirm the existence of GPCR heteromers in brain slices *ex vivo *[35–39].

In situ PLA is based on a pair of antibodies that can bind to target proteins and to which oligonucleotides have been attached. When the so-called proximity probes recognize a target, for example, the receptor heteromer, the attached oligonucleotides are brought into a sufficiently close spatial proximity to allow them to join followed by ligation of the two linear oligonucleotides into a circular DNA molecule. This newly formed DNA circle strand can serve as a template for rolling circle amplification (RCA), resulting in a long single-stranded rolling circle product (RCP) attached to one of the proximity probes. Since the RCP is linked to the proximity probe, it is attached at the site where the proximity probe bound, which means that it can be used to reveal the location of the receptor complex [40, 41]. The RCPs can then be detected and quantified by hybridizing fluorescent oligonucleotides to the repeated sequences of the RCPs, rendering them visible by fluorescence microscopy. With the in situ PLA method the striatal adenosine 2A receptor (A2AR)-dopamine D2 receptor (D2R) heteromers and D2R-oxytocin receptor heteromers have for example, been shown [35, 39]. Also the hippocampal and the mesencephalic raphe FGFR1-5-HT1AR heteromers have recently been demonstrated [36, 37]. The in situ PLA procedure represents a high selectivity and sensitivity assay to demonstrate GPCR heteromers in brain [38]. 

## 6. Allosteric Receptor-Receptor Interactions

**Figure 2:**
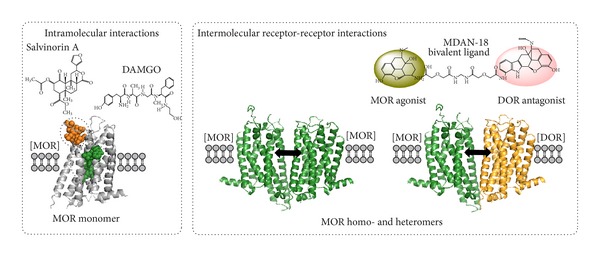
Intra- and intermolecular allosteric receptor-receptor interactions. Allosteric mechanisms make possible the integrative activity taking place intramolecularly in monomers (left) or intermolecularly in homo-/heteromers (right). As one example of the intramolecular allosteric mechanisms is the allosteric binding of salvinorin A to the extracellular site of MOR, which partially affects the activity of the orthosteric MOR binding site via a conformational change [105]. Intermolecular allosteric mechanisms take place through the formation of different types of receptor homo-/heteromers and receptor/protein complexes which can change the function of an individual receptor present in a homomer or heteromer. Another example based on the intermolecular heteromer interactions is the use of heterobivalent ligands containing a MOR agonist and an DOR antagonist linked through a spacer of variable size which may function as useful molecular probes for targeting the MOR-DOR heteromer and in this way counteracting the DOR antagonism on MOR function. Such compounds may have a potential use in pharmacotherapy of pain.

In the beginning, allosteric mechanisms were only discussed in terms of intramolecular interactions within a receptor between orthosteric and allosteric sites. This was the classic pharmacology (Figure 2, left).

Now we have moved into a novel pharmacology, where intermolecular receptor-receptor interactions can occur and results in novel receptor recognition, pharmacology and signaling (Figure 2, right). Intermolecular allosteric mechanisms through the receptor interface produce these changes involving also receptor/protein complexes. An example of the novel pharmacology is the use of heterobivalent ligands [42–44] containing, for example, MOR agonist and DOR antagonist pharmacophores linked through a spacer of variable size which may function as useful molecular probes for targeting the MOR-DOR heteromer and in this way counteracting the DOR antagonism on MOR function [43] (Figure 2, right). Such compounds may have a potential use in pharmacotherapy of pain.

Allosteric mechanism causes a marked rise of the repertoire of GPCR recognition, pharmacology, trafficking and signaling of the participating protomers. This is achieved through changes in recognition, G protein selectivity, and signaling cascades with among others switching from G proteins to *β*-arrestin or to calmodulin [33, 45]. Its function may also change by becoming linked to Receptor Tyrosine Kinases (RTKs) or to ion channel receptors [36, 37, 46].

The term moonlighting protein is used to describe multifunctional proteins in which several functions can be found in a single strand of amino acids unrelated to splicing, posttranslational changes, and so forth [47, 48]. In GPCR heteromers moonlighting is brought about by the allosteric receptor-receptor interactions altering the function of the receptor protomers of the heteromer through conformational changes in single strands of amino acids [46]. 

## 7. The Receptor Interface

We are interested in the receptor interface since it can be a target for novel drugs by their ability to block or mimic the allosteric receptor-receptor interactions [21, 49–51]. The interface in the A2AR-D2R heteromer can be given as an example [50, 51]. It shows helix-helix interactions in the plasma membrane between A2AR TMIV and D2R-TMV. Intracellular electrostatic interactions between D2R IC3 and A2AR C-terminal tail involve positively charged arginines in the D2R IC3 and negatively charged residues in the A2AR especially phosphorylated serine [50–54]. Electrostatic interactions may represent important hot spots in the receptor heteromer interface. The prototype was the A2AR-D2R heteromer but it exists also in the A2AR-D3R and A2AR-D4R interface [55], giving an amazing stability of the heteromers based on the arginine-phosphate bond [54].

Based on a mathematical approach developed by Dr. Tarakanov, we have deduced, based on 48 pairs of receptors that form or not form heterodimers, a set of triplet amino acid homologies that may be critically involved in receptor-receptor interactions [56]. We call it the triplet puzzle. We showed how such triplets of amino acid residues and their “teams” may be used to construct a kind of code that help determine which receptors should or should not form heterodimers. We propose a “guide-and clasp” manner for receptor-receptor interactions where “adhesive guides” may be the triplet homologies [57–60].

The pro-triplet theory has recently became validated [61, 62] underlining its impact on understanding the receptor interface of the heteromers. On the other hand, the proposed contra-triplets, postulated to block the formation of heteromers, still remain to be documented through experimental work. The lack of studies based on the specificities of the established heteromers hamper a proper prediction of the proposed contra-triplets, that now can be optimized through new experimental data.

## 8. On the Existence of MOR, DOR, and KOR and Their Participation in Receptor Heteromers

**Figure 3:**
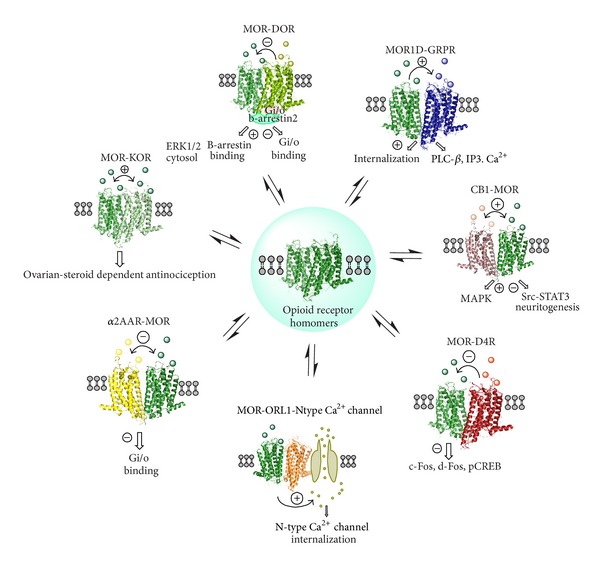
Receptor-receptor interactions in different types of opioid receptor heteromers in the CNS and their potential role in pharmacotherapy of pain. The homo- and heterodimers would allow direct physical interactions between the receptors making possible the allosteric receptor receptor interactions between them. The functional balance between these oligomers determines the final functional output and thus the eventual cellular response. The schematic representation depicts some of the principal, nonexclusive, molecular mechanisms by which opioid heteromers produce novel functions.

The MOR, DOR, and KOR and their heteromers are of special interest since they play a major role in mediating the antinociceptive transmission of the enkephalin and *β*-endorphin neurons in case of MOR and DOR and of the dynorphin neurons in case of KOR (Figure 3). It is known from the fine work of the Watson group that the DOR and especially MOR have a widespread distribution in the brain including the regions of the pain circuits [63]. There exist partial overlaps in the brain and spinal cord of the distribution of the MOR and DOR systems. A widespread distribution is true also for KOR that partially overlaps with the MOR and DOR distribution in the brain and spinal cord. Pain control is all about the balance of activity in the pain and anti-pain systems in the spinal cord, the brainstem, the thalamus, the limbic system and the somato-sensory cortex.

The first opioid receptor heteromer to be discovered was the DOR-KOR heteromer in 1999 by Jordan and Devi [64]. Then in 2000, the MOR-DOR heteromer was demonstrated by George et al. [65] and in 2010 the MOR-KOR heteromer was identified in spinal cord membranes by Chakrabarti et al. [66] and found to be sex specific. They all participate in the modulation of pain.

Together with the existence of opioid heteromers, alternative splicing of the opioid receptor subtypes may help to reconcile the differences between pharmacological subtypes and the results by molecular cloning of only three opioid receptor subtypes. However, also other mechanisms participate [67]. The formation of different types of opioid receptor heteromers through allosteric mechanisms over the receptor interface may contribute to the binding of receptor interacting proteins, producing additional pharmacological subtypes. This can involve, in the latter case, allosteric mechanisms in the receptor-protein interface. 

## 9. MOR-DOR Heteromers and Their Modulation of Pain Circuits

Earlier findings showed that there exist some MOR agonist/DOR antagonist interactions in morphine actions that can be explained by the existence and function of the MOR-DOR heteromer [68], namely, the following.

- Selective DOR blockade with a DOR antagonist reduces the development of morphine tolerance and dependence.
- Chronic administration of morphine results in an upregulation of DORs in rats.
- The intensity of the withdrawal syndrome after chronic morphine treatment correlates with the level of DOR binding sites.
- An antisense oligodeoxynucleotide to DOR was shown to prevent the development of morphine tolerance and dependence after chronic morphine administration [69].
- In DOR knockout mice morphine retained its MOR-mediated analgesic activity without producing tolerance with chronic administration.

All these findings can be explained by DOR exerting an antagonistic allosteric influence on the MOR function in a MOR-DOR heteromer.

In line with the results summarized above, acute *in vitro* experiments on MOR-DOR heteromers in cell lines give evidence that the DOR antagonist enhances MOR recognition, Gi/o coupling and inhibition of cAMP levels. These actions correlated with potentiated morphine analgesia [70].

### 9.1. Hypothesis on the Role of the MOR-DOR Heteromer in Opioid-Induced Tolerance and Dependence

An interesting concept was introduced by Rozenfeld et al. in 2007 [71] to show the difference in the signaling of MOR homomers versus MOR-DOR heteromers upon repeated morphine treatment. You have a mixture of MOR heteromers and homomers in the plasma membrane and the MOR homodimer activation leads to a rapid G protein-mediated ERK1/2 phosphorylation. pERK1/2 goes to the nucleus, where it activates transcription factors contributing to morphine induced analgesia. Instead in the MOR-DOR heterodimer increasing, under chronic morphine treatment, the allosteric mechanism is different. It has switched the coupling from G protein to *β*-arrestin2. You have instead a slow *β*-arrestin2-mediated ERK1/2 phosphorylation. pERK is retained in the cytoplasm and activates cytoplasmic substrates, such as p-p90srk (Ribosomal protein S6 kinase, 90 kDa) with reductions of changes in gene expression and reduction of morphine action [72]. 

Also a time course difference in the MOR homodimer versus MOR-DOR heterodimer mediated ERK phosphorylation in primary dorsal root ganglion neurons was measured. Cotreatment of the heterodimer with a combination of a MOR agonist and a DOR antagonist after chronic treatment with morphine leads through altered allosteric receptor-receptor interaction to a dissociation of *β*-arrestin2 from the heteroreceptor complex and return of the Gi/o binding and coupling to the heteromer and the biological actions of morphine. It may also be associated with a certain disruption of MOR-DOR heteromers into MOR homomers increasing the MOR homomer/MOR-DOR ratio (Figure 3).

### 9.2. Experimental Evidence for Targeting the MOR-DOR Heteromer as a Strategy in Antinociceptive Therapy 

Based on the hypothesis stated above of an increased formation of MOR-DOR heteromers upon chronic morphine treatment contributing to morphine induced tolerance and dependence, novel bivalent compounds with a MOR agonist pharmacophore and DOR antagonist pharmacophore have been developed [43]. Furthermore, opioid-induced tolerance and dependence in mice is modulated by the distance among pharmacophores in a bivalent ligand series, several being substantially more potent than morphine. It offers a new approach for the development of analgesics devoid of tolerance and dependence. One problem with some bridged bivalent compounds is that they may reduce dissociation of the MOR-DOR heteromer and exert a negative allosteric influence on MOR signaling in spite of the block produced by the delta opioid antagonist at the orthosteric site of the DOR.

### 9.3. Effects of Chronic Morphine Treatment on the MOR-DOR Heteromer in the CNS

The above hypothesis is now also supported by new evidence. Gupta and colleagues have found increased abundance of MOR-DOR heteromers after chronic morphine administration [73]. Chronic, but not acute, morphine treatment caused an increase in the abundance of MOR-DOR heteromers in key areas of the CNS that are implicated in pain processing. Because of its distinct signaling properties, the MOR-DOR heteromer may, as outlined above, be a therapeutic target in the treatment of chronic pain and addiction [73].

This fine piece of work was possible through a subtractive immunization strategy to generate antibodies that selectively recognize the endogenous MOR-DOR heteromer but does not recognize either MORs or DORs [74]. Such heteromer specific antibodies may also block or activate the heteromer without influencing the homomer adding to their use as tools in the analysis of the function of these heteromers. Increases could be observed in the rostral raphe region of the medulla oblongata, rich in 5-HT neurons projecting to the dorsal horn, as well as in areas of relevance to reward and mood like ventral tegmental area, nucleus accumbens, prefrontal cortex and hippocampus. 

All the data support a role of this heteromer in morphine tolerance and dependence, since this heteromer develops a reduced G protein-coupling with signaling mainly operating via *β*-arrestin2 (see above). Treatment with DOR antagonists reduces the *β*-arrestin2 coupling in the MOR-DOR heteromer and enhances MOR binding, signaling and morphine-induced antinociception; which may enhance MOR-DOR disruption. It will be of substantial interest to study if acupuncture could favor the formation of MOR homomers versus MOR-DOR heteromers during chronic morphine treatment.

## 10. MOR-KOR Heteromers and Their Involvement in Gender and Ovarian-Steroid Dependent Antinociceptive Actions

The field of MOR-KOR heteromers is also exciting. Formation of MOR-KOR heterodimer is gender-dependent and mediates female-specific opioid analgesia [66] (Figure 3). Spinal morphine antinociception in females, but not males, required the concomitant activation of spinal MOR and KOR. The evidence shows that spinal cord expression of the MOR-KOR heterodimer is sexually dimorphic and dependent on the stage of the estrous cycle. It is elevated in proestrus with high estrogen receptor (ER) levels as seen from coimmunoprecipitation studies obtained with anti KOR antibodies on the spinal cord [66]. 

The evidence also shows that the contribution of dynorphin/KOR (part of MOR-KOR heterodimer) to spinal morphine antinociception is dependent on the stage of the ovarian cycle [66, 75]. Spinal morphine antinociception was quantified using the tail-flick test during diestrus and proestrus. The dynorphin antibody and the KOR antagonist counteracted the morphine-induced anti-nociception in proestrus but not in diestrus. Dynorphin was shown to be linked to the KOR protomer of the MOR-KOR heteromer. It represents a molecular switch that shifts the function of KOR and thereby endogenous dynorphin from pronociceptive to antinociceptive actions. Thus, KOR-MOR heteromer could be a novel molecular target for pain control in women.

Further work has indicated that spinal synthesis of estrogen and concomitant signaling by membrane ER regulate spinal MOR-KOR heterodimerization and female-specific spinal morphine antinociception [76, 77]. There exists coexpression of MOR-IR (immunoreactivity) with ER alpha (ER-alpha), GPR30 (a GPCR for estrogen), or KOR in the superficial dorsal horn. Colocation of MOR-IR and ER-alpha-IR, MOR- and GPR30-IR and MOR-KOR-IR is found in nerve cell bodies and fibers in the superficial dorsal horn. Thus, MOR, KOR, ER-alpha, and GPR30 appear to be coexpressed in neurons of the spinal dorsal horn [76]. 

Biochemical and behavioral experiments suggest that ERs work in a cooperative manner as part of a macromolecular complex to increase KOR/MOR expression. Estradiol (E2) (spinally synthesized and ovarian derived) triggers the formation of a signaling complex that contains multiple ERs and enhances heterodimerization of KOR and MOR. Transcriptional effects of progesterone (P4) are essential either for the formation of the ERs signaling complex and/or the heterodimerization of KOR with MOR [76, 77]. 

In our view the formation of receptor mosaics of activated membrane ER complexes and MOR and/or KOR may take place markedly enhancing the formation of MOR-KOR heteromers in the plasma membrane through allosteric changes in MOR and/or KOR. Possibly, progesterone may contribute through transcriptional increases of opioid receptor interacting proteins that may be essential for the MOR-KOR heteromer formation from the ERs-MOR and/or ERs-KOR heteromer complexes. 

In summary, the gender- and ovarian steroid-dependent recruitment of spinal cord MOR-KOR heterodimers would provide a way to influence the balance between antinociceptive and pronociceptive functions of the spinal dynorphin/KOR opioid system. Impaired formation of MOR-KOR heteromers could be a biological determinant of various types of chronic pain states that are substantially more common in women than men [66]. 

## 11. Opioid Receptor-Like 1 Receptor Heterodimerize with Other Members of the Opioid Receptor Family

The nociceptin receptor or opioid receptor-like receptor 1 (ORL1) belong to the class of Gi/o-linked receptors [78, 79] and is activated by the endogenous 17 amino acid polypeptide ligand orphanin FQ (nociceptin). ORL1 heterodimerize with the other members of the opioid receptor family and can cointernalize each one of them upon agonist exposure.

Upon dimerization with opioid receptors, ORL1 regulation of N-type calcium channels is altered. ORL1 can function as a molecular link that allows MORs to trigger N-type calcium channel internalization [80]. Thus, MOR-ORL1 heterodimers are shown to associate with N-type calcium channels, with activation of MORs triggering N-type channel internalization, but only in the presence of ORL1. Evans et al. [80] found that when coexpressed with the channels alone, ORL1 could trigger internalization of the N-type channels in a nociceptin dose-dependent manner. Without ORL1 expression, activation of MORs by DAMGO did not affect N-type channel surface expression, consistent with a lack of internalization. However, when both MOR and ORL1 were coexpressed, DAMGO application resulted in a dose-dependent loss of N-type channels from the cell surface but not as marked as with ORL1 alone. These findings give the evidence that ORL1 serves as a molecular link allowing MORs to regulate N-type channel surface expression. 

These results are of high interest since opioid and opioid-like receptors play a key role in controlling pain signaling in primary afferent terminals in the dorsal horn by two primary mechanisms [81, 82]. These mechanisms are activation of G protein-coupled inwardly rectifying potassium channels, and inhibition of N-type calcium channels in nerve terminals within the dorsal horn of the spinal cord, both reducing neuronal excitability. In conclusion, formation of opioid/ORL1 heterodimers exerts a profound effect on nociceptive processing [80]. 

Looking at the primary afferents to the dorsal horn clearly activated C and A*δ* nociceptors release a variety of neurotransmitters, activating output neurons in lamina I of the dorsal horn forming part of the pain pathways from the dorsal horn. One important location of the opioid-ORL1 heteromers may therefore be at the central terminals of these nociceptors to inhibit release of transmitters from them involving increased internalization of the N-type Ca^2+^ channels as well as activation of inwardly rectifying K^+^ channels. Again it would be of high interest to explore how acupuncture would modulate the formation of these receptor heteromeric complexes in the dorsal horn.

## 12. Do the Alpha-2A Adrenergic Receptor-MOR Heteromers Have a Role in Pain Processing Pathways? 

Early work indicates that agonists acting at the alpha-2A adrenergic receptor subtype (alpha-2AAR) and opioid receptors have analgesic properties and act synergistically when co-administered in the spinal cord. The alpha-2AAR subtype is the primary mediator of alpha2 adrenergic spinal analgesia and is necessary for analgesic synergy with opioids and feedback inhibition of capsaicin-induced hyperalgesia [83, 84]. Other findings also demonstrated that alpha-2AAR potentiated morphine analgesia. Thus, a mutual potentiation of anti-nociceptive effects of morphine (opioid agonist) and clonidine (alpha-2AAR agonist) was demonstrated between the antinociceptive effects of intrathecal clonidine and systemic morphine which may be effective in the treatment of chronic pain states [85–87]. 

The question is if alpha-2AAR-MOR heteromers can participate in these synergistic actions? The major origin of NA innervation of the dorsal horn by the descending bulbo-spinal NA systems [10, 12] is the locus coeruleus (LC) [11]. NA or clonidine significantly reduces the evoked release of glutamate from spinal cord synaptosomes [88] and the release of substance P (SP)-like material and calcitonin gene related peptides (CGRP) from spinal cord slices [89]. Such actions could explain the antinociceptive actions of alpha-2AAR activation. 

Immunoreactivity for both alpha-2AAR and MOR is observed in the superficial layers of the dorsal horn of the spinal cord. The primary localization of the alpha-2AAR in the rat spinal cord is on the terminals of capsaicin-sensitive, SP-containing primary afferent fibers (colocation with MOR IR). Thus, alpha-2AAR-MOR heteromers may exist on these terminals.

The role of the receptor-receptor interactions in the alpha-2AAR and MOR heteromers was found to be an unexpected one [90]. There exists a conformational antagonistic crosstalk between alpha-2AAR and MORs in their control of cell signaling upon coactivation (Figure 3). Activation of MOR by morphine modulates alpha-2AAR signaling by a direct strong antagonistic conformational change that propagates from MOR to alpha-2AAR within 0.4 s. The inhibition of Gi activation in the reverse direction also suggests a conformational propagation from alpha-2AAR to MOR. The conformational spread conveyed by the two agonists, noradrenaline and morphine leads to functional inhibition upon agonist coactivation, called cross-inhibition [90]. This is likely a means of rapidly preventing overstimulation of the same signaling pathway as also discussed for alpha-2AAR-Neuropeptide Y receptor (NPY receptor) interactions [91], which results in a cross-inhibition of alpha-2AAR and NPY receptors, both coupled to Gi/o, in biochemical and functional studies on vasodepressor responses. These studies may serve as a model for understanding fast desensitization mechanisms in several signaling pathways. These results suggest that combined agonist activation of alpha-2AAR-MOR heteromers could play a role in counteracting excessive analgesia.

It follows that synergy of alpha-2AAR agonist and morphine in antinociception cannot be explained by receptor-receptor interactions in the alpha-2AAR-MOR heteromers. In this case, interactions at the level of signaling pathways and ion channels controlled by the corresponding homomers may be involved as well as a location in different nerve cells of the neuronal network synergizing in favoring an output pathway leading to antinociceptive effects.

## 13. On the Localization and Functional Roles of Cannabinoid CB1 Receptors in Pain-Processing Pathways

Synergistic interactions also exist between cannabinoid and opioid analgesia [92]. The cannabinoid CB1 receptors (CB1) are activated by the endocannabinoids, 2-arachidonoylglycerol (2-AG) and anandamide which are recognized for mediating retrograde signaling at glutamate and GABA synapses mediating depression of depolarization induced suppression of excitation and of inhibition, respectively [93]. They likely communicate via an extracellular vesicle mediated form of VT [94, 95]. They can be formed by budding from lipid rafts (shedding vesicles) and may impinge on the plasma membrane of target cells to transfer lipid rafts with associated receptor oligomeric complexes and lipid messengers like the endocannabinoids.

CB1s are present at many locations in the pain networks namely in peripheral terminals of primary sensory neurons, at synapses in the spinal cord, and in pain circuits of the brain. At spinal synapses, CB1 could be on nerve terminals of afferent neurons, on interneurons, and/or on terminals of pathways originating in supraspinal regions [96]. 

A strong colocalization of CB1 and MOR has been observed in lamina II interneurons [97]. The CB1 action can also involve the inhibition of N-type Ca^2+^ channels and activation of inwardly rectifying K^+^ channels in afferent terminals and dorsal horn neurons as discussed for the alpha-2AAR and MORs.

## 14. On the Role of CB1-MOR Heteromers for Neuroplasticity in Pain Pathways

Rios et al. in 2006 demonstrated that CB1 forms heteromers with opioid receptors [98]. A BRET signal is formed with MOR, DOR and KOR in cellular models. Thus, coexpression of opioid receptors with CB1, but not with chemokine receptors, leads to a significant increase in the level of BRET signal giving evidence for the existence of CB1-opioid receptor heteromers (Figure 3).

Simultaneous activation of MOR and CB1 leads to a significant attenuation of the increase in MAPK phosphorylation response seen upon activation of the individual protomers. Thus, upon agonist coactivation antagonistic receptor-receptor interactions develop in the CB1-MOR heteromers as observed for the alpha-2AAR-MOR heteromers. However, when the CB1 protomer alone is activated in the CB1-MOR heteromer there is a marked increase in signaling compared with the agonist activation of CB1 monomer/homomer.

Similar results were obtained on neurite outgrowth in Neuro-2A cells expressing MOR and CB1 's. Agonist-induced neurite outgrowth in Neuro-2A cells treated with a combination of 100 nM DAMGO or morphine, and 100 nM CB1 agonist HU-210 is markedly reduced upon coactivation while increased with single agonist activation. Upon coactivation a substantial cross-inhibition of the phosphorylation of Src and STAT3 is observed [98].

Antagonistic allosteric interactions in CB1-MOR heteromers may underlie the attenuation of the Src-STAT3 pathway signaling which could be one of the mechanisms leading to reduction of neurite outgrowth. MOR-CB1 interactions will thus upon coactivation lead to cross-inhibition of neuritogenesis involving inhibition of the Src-STAT3 pathway. Such a phenomenon may be of substantial importance. Thus, it may lead to counteraction of the plasticity changes seen in discrete pain networks leading to chronic pain [99]. If so, coactivation of MOR and CB1 is the way to go since single activation of the protomers leads to increases in plasticity [98]. It should be considered that RTK can also be involved in these plasticity responses forming a heterotrimer with the CB1-MOR heterodimer making possible integration of transmission and trophic signaling already at the plasma membrane level [8, 36, 37, 100]. 

## 15. On the Existence of CB1-D2R and D4R-MOR Heteromers and Their Role in Addiction

There exist indications for the existence also of a CB1-D2R heteromer in which A2AR may participate [101, 102] in the striatopallidal GABA neurons, a key pathway in reward mechanisms. D2R play a major role in cocaine addiction development where the receptor-receptor interactions in CB1-D2R heteromers may exert beneficial actions. During a state of dominance of D2R activation, a negative-feedback regulation of D2R remains through the D2R-mediated release of anandamide inducing an antagonistic CB1-D2R interaction which counteract the exaggerated activation of the D2Rs. In this heteromer, through allosteric receptor-receptor interactions, CB1 may also become coupled to Gs [101, 103] to reduce the downstate induced by the excessive D2R activation, contributing to addiction development.

There may also exist D4R-MOR heteromers in the nucleus accumbens and dorsal striatum of relevance to the treatment of addiction (Figure 3). Thus, D4R activation decreases MOR IR in the striatal islands [104] and D4R can modulate the affinity of MOR in striatum. Furthermore, D4R activation counteracts the morphine induced increases in the striatal expression of the transcription factors c-Fos, deltaFosB and P-CREB [105]. These results can be explained on the existence of antagonistic D4R-MOR interactions in striatal D4R-MOR heteromers [106]. D4R-D2R heteromers [107] and D4R homomers [108] have previously been shown to exist. It will be of interest to evaluate if acupuncture treatment in drug addiction can modulate the striatal CB1-D2R and D4R-MOR heteromers and their antagonistic receptor-receptor interactions.

## 16. MOR Isoform 1D (MOR1D) Heterodimerizes with Gastrin-Releasing Peptide Receptor (GRPR) in the Spinal Cord: A Key Role in Morphine Induced Itch

In 1983 we discovered neuronal gastrin releasing peptide (GRP) IR in the rat CNS [109]. In the revision we were forced to add also bombesin like IR in the description of the IR since the reviewer claimed it was not possible to fully exclude this possibility. GRP was discovered in porcine nonantral gastric tissue [110]. C-terminal specific antisynthetic porcine GRP sera R-6902 and R-6903 were used showing GRP-like IR in brain tissue extracts. As control was used an antibombesin (BN) serum with the major immunological determinant residing in the 6-7 peptide sequence of BN which is lacking GRP. The results favored the existence of GRP-like IR terminals especially found in the marginal layer and in the substantia gelatinosa of the dorsal horn having a codistribution with SP IR terminals.

Sun and Chen discovered that GRPR mediates the itch sensation in the spinal cord which brought GRP transmission into the spotlight [111]. In 2011 Liu et al.'s group [112] demonstrated coexpression of GRPR and the MOR isofom MOR1D in Lamina I of the spinal cord but not with the MOR1 isoform.

MOR1D was shown to heterodimerize with GRPR in the spinal cord, relaying itch information [112]. Spinal opiates were found to produce itch through MOR1D-GRPR heteromerization leading to cross activation of GRPR signaling (PLC-*β*/IP3-dependent Ca^2+^ signaling pathway) (Figure 3). They showed that morphine triggers internalization of both GRPR and MOR1D, while GRP specifically triggers GRPR internalization and morphine-independent scratching [112].

The data suggest that opioid-induced itch is an active process concomitant with but independent of opioid analgesia, occurring via the unidirectional cross-activation of GRPR signaling by MOR1D-GRPR heterodimerization. The evidence demonstrates that the C-Terminus of the MOR1D is critical for the MOR1D-GRPR heterodimer formation. The difference between MOR1 and MOR1D isoforms lies in a motif consisting of seven amino acids (RNEEPSS) located in the C-terminus of the MOR1D.

To test the spinal functions of the heteromer, a Tat-fusion peptide (Tat-MOR1D CT) was synthesized. The Tat-motif (YGRKKRRQRRR) belong to a trans-activating domain of a HIV protein that can permeate the cell membrane allowing, after intrathecally injection into the spinal cord, the introduction of the fused MORD1 C-terminal (RNEEPSS motif) into the cells. Introduction of the Tat-MOR1D CT permits its competition with MOR1D for physical contacts with GRPR *in vivo.* It specifically blocked morphine induced scratches while leaving GRP induced scratches intact, morphine induced analgesia unaltered and a reduction in the coimmunoprecipitation of MOR1D-GRPR levels.

New insights into opioid-induced itch prevention was in this way obtained. They also demonstrated that molecular and pharmacologic inhibition of PLC-*β*3 and IP3R3, two downstream effectors of GRPR, specifically blocked morphine induced scratches but not morphine induced analgesia [112]. Based on these observations it would be of high interest to test if acupuncture can counteract the formation of the MOR1D-GRPR heteromers. This would give a biological basis for its use in treatment of itch.

## 17. Nociceptors in the Peripheral Nervous System and Their TRPA1-TRPV1 Heteromeric Complexes

Of high interest are the peripheral nociceptors and their Transient receptor potential cation channel, subfamily A, member 1 (TRPA1)-Transient receptor potential cation channel, subfamily V, member 1 (TRPV1) heteromeric complexes. TRPV1, one heat nociceptor, is the most famous one, since it represents the capsaicin or vanilloid receptor, activated by ingredients in “hot” chili peppers [7]. TRPA1 is a chemical nociceptor. It is a receptor for pungent ingredients in mustard and garlic plants, isothiocyanates and thiosulfinates. These nociceptor terminals also express a host of sodium channels and potassium channels (such as TRAAK and TREK-1) that modulate nociceptor excitability and/or contribute to action potential propagation [7]. The capsaicin TRPV1 is a nonselective cation channel that is structurally related to members of the TRP family of ion channels [113]. The membrane topology and domain structure of TRPV1 have been predicted. TRPV1 ion channel has high Ca^2+^ permeability and the capsaicin activation of this channel kills the cells.

By means of acceptor bleaching FRET, a direct interaction between TRPA1 and TRPV1 on the plasma membrane was observed [114]. The increase in donor emission between TRPA1 and TRPV1 in the plasma membrane is just as large as between the corresponding homomers TRPA1-TRPA1 and TRPV1-TRPV1 as has been demonstrated by measurement of the FRET signal efficiency.

Mustard oil activates single channel currents in TRPA1 and TRPA1-TRPV1 expressing CHO cells. Vigorous activation of channels in TRPA1 and TRPA1-TRPV1 expressing cells was observed but not in untransfected CHO cells with multiple conductance states, performed in cell-attached configuration in voltage clamp mode [114]. However, differences of the current voltage relationships have been found in the single channel activities of TRPA1 alone and TRPA1-TRPV1 heteromers. The single channel mustard oil induced conductance (IMO) current-voltage I-V relationships for TRPA1-containing cells showed hardly any rectification. In contrast, the TRPA1-TRPV1 channel heteromers resulted in an outward rectification with a high conductance slope, for the outward versus the inward parts of the I-V curve, respectively. Thus, the ion channel function becomes altered through the TRPA1-TRPV1 heteromerization. Also, results on the properties of single channel mustard oil induced conductances (IMO) in TRPA1 in WT and TRPV1 KO sensory neurons validated these findings. IMO exhibited substantially greater activity at positive voltages in WT neurons compared with TRPV1 KO neurons [114]. 

These results support the hypothesis that TRPV1 and TRPA1 in nociceptors may form a heteromeric receptor ion channel complex and that TRPV1 can influence intrinsic characteristics of the TRPA1 channel also independent of intracellular calcium [114, 115]. Transmission of inflammatory stimuli by nociceptors (namely damage-sensing sensory neurons) is also mutually controlled by TRPA1 and TRPV1 channels. This functional interaction between TRPV1 and TRPA1 could occur indirectly via recruitment of second messengers, such as intracellular Ca^2+^ and/or directly, involving allosteric receptor-receptor interactions between these receptor channels within a heteromeric complex.

The demonstrated pharmacological cross-desensitization between capsaicin and mustard oil responses can involve desensitization of TRPA1 and TRPV1 ion channel activities in their heteromeric complexes which may contribute to inhibition of nociceptor signaling leading to antihyperalgesia and antinociception [114, 115]. One mechanism for the antinociceptive actions of acupuncture along meridians may be that it enhances the cross-desensitization of the TRPA1-TRPV1 heteromeric complexes within the nociceptors located along these meridians. This process may inter alia involve changes in the flow of volume transmission signals in channels containing extracellular fluid and nociceptors along the meridians modulating the sensitivity of the nociceptors [22, 82].

## 18. Ionotropic Cannabinoid Receptors in Peripheral Antinociception and Antihyperalgesia

There is also a role of ionotropic cannabinoid receptors (ICR) in peripheral antinociception and antihyperalgesia. The known ICRs are members of the family of transient receptor potential channels (TRP) and remarkably include TRPV1, TRPV2, TRPV4, TRPM8, and TRPA1 (see above). The majority of ICRs are expressed in nociceptive sensory neurons, which can detect and respond to noxious mechanical, thermal and chemical stimuli. Nevertheless, the cannabinoids produce a profound antihyperalgesia and the mechanism has not yet been established [115]. 

One possible hypothesis addressing this issue is that partial activation of ICRs does not necessarily generate excitation (i.e., action potential) of nociceptors. From this perspective, it is interesting that cannabinoids are not full agonists for TRP channels. Indeed, cannabinoids typically evoke a slow generation of small inward currents and Ca^2+^ accumulation. As a result, cannabinoid-gated responses might not reach the threshold levels required to excite nociceptors. Moreover, slow depolarization of nociceptor membrane potentials might lead to inactivation of voltage gated channels that, in turn, inhibits the generation of action potentials.

To understand how activation of ICRs leads to inhibition of nociceptors, molecular mechanisms of desensitization of TRP channels by ICR-activating cannabinoids have been studied [115]. The results indicate that cross desensitization between the TRPA1 and TRPV1 channels (see also above) in sensory neurons can involve multiple separate mechanisms. Cannabinoids may desensitize TRPV1 channels via activation of calcineurin and dephosphorylation of the ion channel. Homologous desensitization of TRPV1 can occur by application of TRPV1-selective cannabinoids, and heterologous desensitization of TRPV1 can occur by administration of TRPA1-selective cannabinoids (e.g., WIN55212). Cannabinoids can also desensitize TRPA1 via activation of a calcium-independent pathway. 

Based on the existence of TRPA1-TRPV1 heteromeric complexes we may also hypothesize the following mechanism: Ionotropic cannabinoids can activate antagonistic allosteric channel-channel interactions in such types of heteromeric complexes. This allosteric mechanism especially upon coactivation of the two TRP channels by selective ionotropic cannabinoids may produce cross-desensitization of the two nociceptor channels leading to antinociception and antihyperalgesia. The ionotropic cannabinoids may best be regarded as negative allosteric modulators of TRPA1-TRPV1 heteromeric complexes and other types of TRP heteromeric complexes. It is presently unknown to which extent metabotropic CB1 and CB2 may participate in the modulation of the peripheral nociceptors and in the mediation of the actions of the ionotropic cannabinoids. A dynamic interplay between CB1/CB2 and TRP channels as to heteromerization is however, an interesting possibility.

Other mechanisms for acupuncture induced analgesia likely also exist since analgesic effects also develop in distant parts of the body. There exists a mechanism called diffuse noxious inhibitory control (DNIC) which results in reduction of pain from the experimental noxious stimulus when a nociceptive stimulus is applied to a region remote to the test area [20, 116, 117]. DNIC has inter alia been shown to be activated in experimental peripheral mononeuropathy [118] where peripheral mechanisms may mainly be involved like sensitization of damaged nerve fibers. However, acupuncture can activate pathways involved in DNIC as found in studies on trigeminal caudalis neurons in rats [119]. However, lower pain intensities are used in human acupuncture which may explain why the analgesic effects of acupuncture in humans are less than a DNIC effect of a painful noninvasive stimulus [116].

It should also be noted that sensory neuropeptides like substance P may be released from nociceptors upon acupuncture and both acupuncture and sensory neuropeptides increase cutaneous blood flow [120]. It is of particular interest that somatostatin peptides may produce systemic analgesic effects [121]. Thus, somatostatin can produce inhibition of the cross-excitation between adjacent primary afferent terminals in rats induced by antidromic stimulation of primary afferents leading to inhibition of peripheral hyperalgesia. Therefore, one additional mechanism for acupuncture analgesia should be considered namely that somatostatin can be released by acupuncture from sensory nerve terminals along the meridians. It may then diffuse and flow along the interstitial fluid channels of the meridians for short (extrasynaptic mode) and/or long distances of volume transmission [122] to activate somatostatin receptors located on the plasma membrane of the nociceptors to reduce their firing and produce reduction of pain. The findings of Guo et al. (2008) [121] indicate that released somatostatin may also use the surrounding vascular beds to reach via the circulation adjacent primary afferents and also via this mode of communication produce peripheral analgesic effects via activation of somatostatin receptors.

## 19. Future Directions

The role of the receptor heteromers in pain modulation may be studied along the following lines.

- Understanding the role of MOR-DOR, MOR-KOR, alpha-2AAR-MOR, and CB1-MOR heteromers in key pain circuits in the CNS. Of special interest will be to outline the role of opioid receptor heteromers as a target for the treatment of pain including acupuncture and the role of MOR1D-GRPR heteromer in itch and as a target for the anti-pruritus actions of acupuncture.
- Understanding the role of TRPA1-TRPV1 heteromeric ion channel complexes in nociceptor function and their role in the antinociceptive and antihyperalgesic actions of cannabinoids.
- Characterization of the receptor interfaces in distinct opioid receptor and TRPA1-TRPV1 heteromers and putative ERs-MOR-KOR heteromeric complexes. The receptor interface is a novel target enabling modulation of the allosteric receptor-receptor interactions.
- Understanding the pharmacology of the above receptor heteromers. A major targets for the therapeutic effects of antinociceptive drugs which may mediate side effects.
- Understanding the link of potential changes in distinct opioid receptor and TRPA1-TRPV1 heteromer structure and function to plasticity changes in the pain pathways in chronic pain syndromes. Discovery of novel key receptor heteromers is still to come.
- Finally, discrete heteromers may also be targets for chemical ingredients mediating the medicinal properties and the side-effects of plants that is, herbal medicine.

## Glossary

 The usage of terms in medicine often varies widely. For this reason, it is convenient and helpful to authors and readers if words can be used with an agreement in their technical meaning. The definition provided in this Glossary are intended to be specific andexplanatory and to serve as a useful framework, not as a constraint on feature development for members who work in the field of pain or are interested in this review article topics (all definitions are taken from Andreas Kopf Guide Pain Management in Low-Resource Settings, International Association for the Study of Pain).

*Acupuncture*. Acupuncture is a procedure involving the stimulation or inhibition at ananatomical location on or in the skin by a variety of techniques. A number of effects on painphysiology have been identified, the most important being the activation of the endogenousopioid system and the spinal modulation of pain signaling through activation of touch fibers (A*β* fibers).

*Analgesia*. Absence of pain in response to stimulation that would normally be painful. The stimulus is defined by its usual subjective effects.

*Hyperalgesia*. An increased response to a stimulus that in normally painful. Hyperalgesia reflects increased pain on supra-threshold stimulation. For pain evoked by stimuli that usuallyare not painful, the term allodynia is preferred, while hyperalgesia is more appropriately usedfor cases with an increased response at a normal threshold, or at an increased threshold, such asin patients with neuropathy. It should also be recognized that with allodynia the stimulus andthe response are in different modes, whereas with hyperalgesia they are in the same mode.

*Meridian*. In the Chinese medicine acupuncture means each of a set of pathways in thebody along which vital energy is said to flow. There are twelve such pathways associated with specific organs.

*Nociception*. Nociception is the sensory component of pain. It encompasses the peripheral and the central neuronal events following the transduction of damaging mechanical, chemicalor thermal stimulation of sensory neurons (nociceptors).

*Nociceptor*. A receptor preferentially sensitive to a noxious stimulus or to a stimulus thatwould become noxious if prolonged. Often called a pain receptor.

*Noxious Stimulus*. A noxious stimulus is one that is damaging to normal tissue.
